# Th1-related transcription factors and cytokines in systemic lupus erythematosus

**DOI:** 10.3389/fimmu.2023.1305590

**Published:** 2023-12-18

**Authors:** Yang-Yang Tang, Da-Cheng Wang, You-Yue Chen, Wang-Dong Xu, An-Fang Huang

**Affiliations:** ^1^Department of Evidence-Based Medicine, Southwest Medical University, Luzhou, Sichuan, China; ^2^Department of Rheumatology and Immunology, Affiliated Hospital of Southwest Medical University, Luzhou, Sichuan, China

**Keywords:** Th1 cell, T-bet, IL-2, lupus, inflammatory cytokines

## Abstract

Systemic lupus erythematosus (SLE) is an inflammatory disorder related to immunity dysfunction. The Th1 cell family including Th1 cells, transcription factor T-bet, and related cytokines IFNγ, TNFα, IL-2, IL-18, TGF-β, and IL-12 have been widely discussed in autoimmunity, such as SLE. In this review, we will comprehensively discuss the expression profile of the Th1 cell family in both SLE patients and animal models and clarify how the family members are involved in lupus development. Interestingly, T-bet-related age-associated B cells (ABCs) and low-dose IL-2 treatment in lupus were emergently discussed as well. Collection of the evidence will better understand the roles of the Th1 cell family in lupus pathogenesis, especially targeting IL-2 in lupus.

## 1 Introduction

Systemic lupus erythematosus (SLE) is a complex inflammatory autoimmune disease, characterized by multiple organs and system damage during relapse and remission periods. This disease mainly occurs in women, especially those of childbearing age. To date, the pathogenesis of SLE has not yet been completely elucidated. Numerous studies showed that a pathological hallmark of SLE is disorder of the regulatory function in the immune system. There are altered immune responses to autoantigens with autoantibody generation, apoptotic cell clearance deficiency, and tissue injury mediated by deposition of the immune complex. Lupus nephritis (LN) is a frequent and serious complication of SLE, characterized by proteinuria, heterogeneous histologic alteration, and massive deposition of immune complexes in kidneys.

Both innate and adaptive immune systems select kinds of mechanisms to orchestrate the delicate balance between regulatory and effector cells so as to prevent deleterious activation. There is an abnormal crosstalk between different immune cells such as B cells and T helper 1 (Th1) cells, Th1 cells, and Th2 cells in SLE. CD4^+^ Th cells can be divided into Th1, Th2, Th17, and regulatory T (Treg) cells. Differentiation of different Th cells depends on the local cytokine milieu and co-stimulation provided by antigen-presenting cells. Th1 cells and Th2 cells are able to express their key cytokine interferon γ (IFNγ) and interleukin-4 (IL-4), respectively. Transcription factor T-box expressed in T cells (T-bet) and GATA binding protein 3 (GATA3) individually regulate Th1 cell and Th2 cell differentiation by promoting the production of IFNγ and IL-4. Th17 cells have the ability to generate IL-17A, mediated by transcription factor retinoic acid receptor-related orphan receptor-gammat (RORγt). Treg cells are considered as suppressive effector T cells, which regulate autoreactive T-cell responses. Distinct Th cells play different roles in a series of events culminating in systemic tissue damage in lupus. For example, there was much infiltration of inflammatory Th cells in kidneys of LN patients and lupus-prone mice, indicating evidence of recent activation of the Th cells. Moreover, activated autoreactive T cells in lupus presented with aberrant T-cell signaling and co-stimulation and Treg cell dysfunction. In recent years, Th1 cells and related cytokines are recognized to strongly correlate with SLE pathogenesis, especially the newly identified roles of the transcription factor T-bet and cytokines such as IL-2. Therefore, in this review, we comprehensively discussed potential roles of Th1 cells, T-bet, cytokines IFNγ, tumor necrosis factor alpha (TNFα), IL-2, IL-18, transforming growth factor-beta (TGF-β), and IL-12 in autoimmune lupus. Collection of the information will help to better understand the roles of Th1 cells and related cytokines in lupus development.

## 2 Th1 cells

Th1 cells play a major role in lupus pathogenesis and has gained wide interest as a treatment target. In studies from a Tunisian population and a Northern Chinese population, SLE patients treated with glucocorticoids and hydroxychloroquine showed a higher percentage of CD4^+^IFNγ^+^ (Th1) cells as compared with that in healthy controls (1, 2). SLE patients showed a higher expression of P2X7 receptor (P2X7R) on Th1 cells than healthy controls (2). On the contrary, SLE patients from Southern China and Germany treated with prednisone, azathioprine, mycophenolate mofetil, and methotrexate revealed a lower percentage of Th1 cells than healthy controls (3, 4). Interestingly, a study from North American SLE patients treated with methotrexate, azathioprine, and cyclophosphamide found that there was a comparable frequency of Th1 cells between SLE patients and healthy controls (5). Another study of Japanese SLE patients treated with several immunosuppressants (cyclophosphamide, azathioprine, tacrolimus, cyclosporin, methotrexate, mizoribine, rituximab, hydroxychloroquine, abatacept, mycophenolate mofetil, corticosteroids) suggested that SLE patients had higher percentages of CD4^+^CXCR3^lo^T-bet^hi^ cells, CD4^+^HLA-DR^+^CD38^+^CXCR3^lo^T-bet^hi^ cells, and CD45RA^+^Foxp3^lo^IFNγ-producing-T-bet^+^ cells and phosphorylation expression of mammalian target of rapamycin (mTOR) in peripheral CD4^+^ T cells than those in controls (6). The phosphorylation expression of mTOR was related to T-bet expression in CXCR3^lo^T-bet^hi^CD4^+^ cells, and CXCR3^lo^T-bet^hi^ cells had a high expression of IFNγ in SLE patients. Similarly, German SLE patients treated with immunosuppressive agents (prednisone, hydroxychloroquine, azathioprine, mycophenolate mofetil, belimumab) with active disease activity reported a higher expression of B- and T-lymphocyte attenuator (BTLA) in CD3^+^CD8^-^CD69^+^IFNγ^+^ cells than that in inactive SLE patients and healthy controls (7). Furthermore, there was a higher proportion of CD3^+^IFNγ^+^ cells in class IV LN patients (without treatment) than controls, correlating with the histologic activity index (8). This was demonstrated in a study that class I, II, III, or IV LN patients after treatment with prednisolone showed elevated expression of anti-dsDNA antibody, increased number of Th1 cells as compared with healthy controls, and most of the Th1 cells shown by IL-12 receptor-positive (IL-12R^+^) T cells (9). CD3^+^T-bet^+^ cells were found in the glomeruli and interstice of all LN patients, correlating with urinary Th1 cells (10). SLE patients also showed increased expression of monocyte chemoattractant protein 1 (MCP-1) and interferon protein-10 (IP-10) in all LN patients, indicating that a higher expression of chemokines may transfer Th1 cells into gromeluri (9). Collectively, the above findings showed that SLE patients (LN patients) under different treatments may have different percentages and numbers of Th1 cells. Interestingly, relationships between Th1/T-bet aspects and type I interferon are interesting immunity. Memory CD45RO^+^ T cells from peripheral blood mononuclear cells (PBMCs) of patients with active Behçet disease (BD) and SLE and healthy controls were stimulated with IFNα, showing an elevated expression of IFNγ in memory CD4^+^ T cells in BD patients and controls, but not in SLE patients (11). IFNα stimulation also upregulated IL-10 synthesis in memory Th1 cells, suggesting that IFNα promoted a regulatory Th1 response by IL-10 secretion in BD (11). Plasmacytoid dendritic cells (pDCs) from SLE patients treated with prostaglandin E2 (PGE_2_) inhibited the expression of IFNα, and coculturing PGE_2_-treated pDCs with Th1 cells significantly suppressed secretion of IFNγ, indicating that IFNα is involved in PGE_2_-mediated inhibition of Th1 response (12). Furthermore, childhood-onset SLE (cSLE) patients showed a high expression of IFNα and IFNγ as compared with controls, and IFNγ stimulation induced expression of T-bet in B cells (13).

Since Th1 cells were aberrantly expressed in lupus patients, it is necessary to understand more about the regulation of Th1 cell differentiation. When compared with control mice, the expression of PTEN-induced kinase 1 (Pink1) in CD4^+^ T cells from MRL/lpr lupus mice was decreased (14). Naive CD4^+^ T cells from Pink1-deficient (Pink1^−/−^) mice treated with Th1-polarizing conditions had a higher proportion of Th1 cells than that from wild-type (WT) mice (14). In the absence of Th1-polarizing conditions, CD4^+^ T cells from Axl and Mertk double-knockout mice generated higher levels of IFNγ and TGF-β than those of WT mice (15). Upon stimulation by anti-CD3 and anti-CD28 antibodies, there was an elevated percentage of CD4^+^IFNγ-producing-T cells in Axl and Mertk double-knockout mice. When CD4^+^ T cells from Axl and Mertk double-knockout mice were treated with Th1-polarizing conditions, the expression of IL-12 was increased. However, addition of neutralization with the anti-IL-12 antibody or anti-IL-18 antibody downregulated IFNγ production (15). After adoptively transferring IL-2-stimulated CD4^+^ɑβ Th1 clones (dna51 (Vβ8.3) and rnp2 (Vβ14)) to MRL/lpr lupus mice or transferring irradiated CD4^+^ɑβ Th1 clones to lupus mice, serum levels of anti-dsDNA antibody, activity index for LN, and numbers of CD4^+^Vβ8.3 T cells in spleen were reduced and dna51 cell proliferation and cytotoxicity of CD8^+^ T cells against dna51 were inhibited as well (16). Interestingly, there were anti-idiotypic antibodies recognizing a 12 amino acid sequence of clone dna51 T-cell receptor Vβ8.3-complementarity-determining region 3 in the treated MRL/lpr mice. Moreover, both lipocalin-2 (LCN2) expression in CD4^+^ T cells from LN patients, LCN2 expression in peripheral blood mononuclear cells (PBMCs), and splenic CD4^+^ T cells from MRL/lpr mice were higher than those in controls, correlating with SLE disease activity index and urine protein and serum creatinine expression (15). Injection of the recombinant LCN2 protein into MRL/lpr mice promoted Th1 cell differentiation in spleen and lymph node, whereas administration of the anti-LCN2 antibody in MRL/lpr mice downregulated the percentage of Th1 cells. CD4^+^LCN2^−/−^ T cells under Th1-polarizing conditions differentiated less into Th1 cells, along with low expression of IFNγ, IL-12Rβ2, of T-bet. Addition of recombinant LCN2 upregulated Th1 cell differentiation. When LCN2^−/−^ mice were injected with pristane to induce lupus, there were reduced glomerular IgG and C3 deposits, macrophage and neutrophil infiltration, and less frequency and number of Th1 cells (15). Therefore, Pink1, LCN2, Axl, and Mertk regulate Th1 cell differentiation and proliferation, where deficiency of Pink1, double-knockout Axl, and Mertk promote differentiation of Th1 cells, LCN2 exacerbates lupus by promoting Th1 cell differentiation, and CD4^+^αβ Th1 clone vaccination elicits anti-idiotypic T-cell responses in lupus.

## 3 Transcription factor T-bet

### 3.1 Association of T-bet and SLE

**Figure 1:**
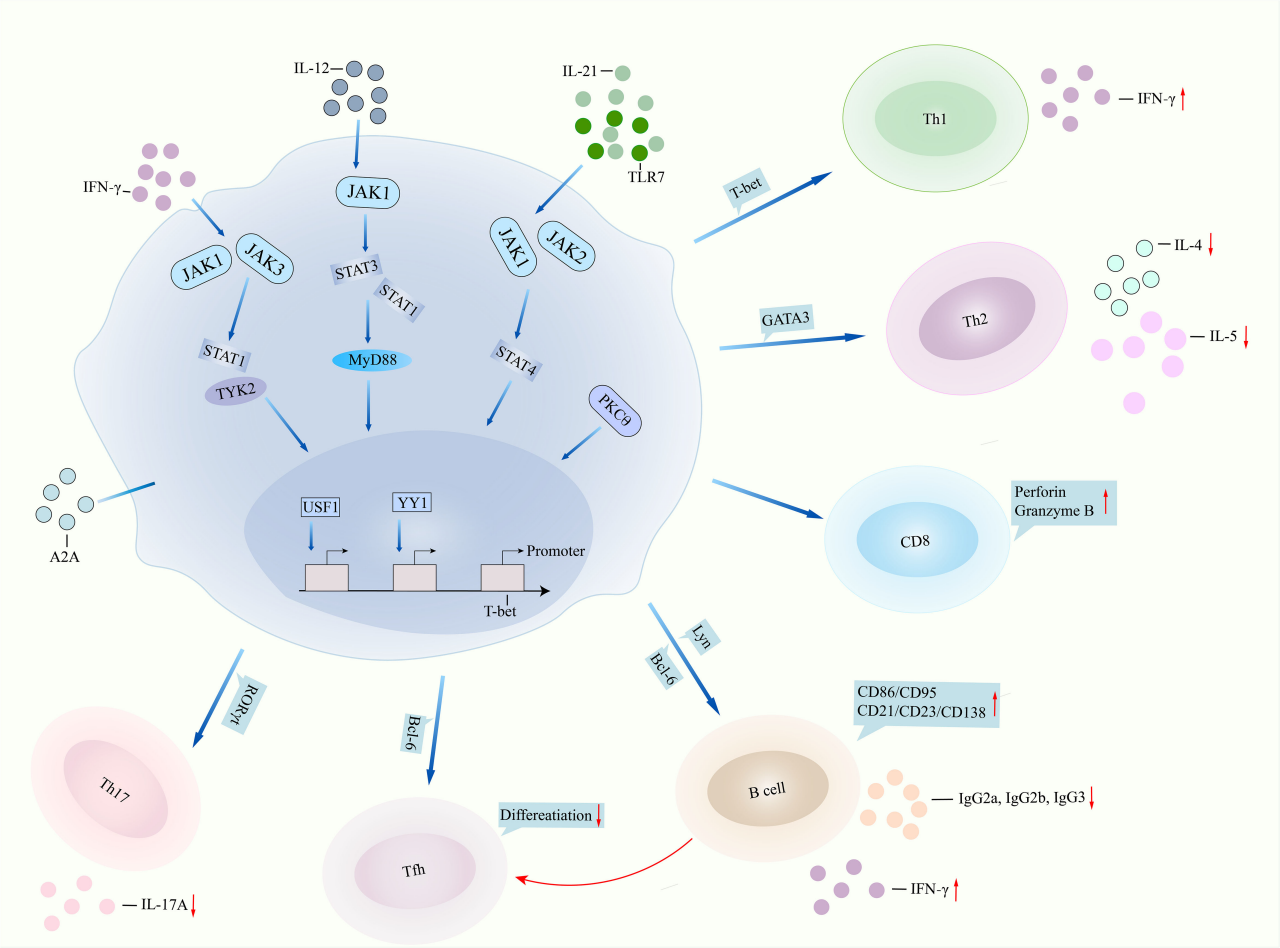
T-bet involved in lupus pathogenesis. T-bet performs in lupus pathogenesis by two pathways, namely, dysfunction of itself and aberrant interaction with other immune cells. IFNγ binds to JAK1/JAK3 and then regulates STAT1/TYK2 signalings, leading to overactivation of T-bet, or TLR7/IL-21 binds to JAK1/JAK2/STAT4 signalings and then results in T-bet overexpression. IL-12 can also bind to JAK1/STAT1/STAT3 signaling and finally regulate T-bet function. Transcription factors USF1 and YY1 repressed the promoter expression of the T-bet gene. Deficiency of T-bet in CD4^+^ T cells inhibits the expression of IFNγ, leading to suppression of Th1 cell differentiation but promotion of Th2 cell activity. T-bet binds to the promoter of perforin and granzyme B, regulating cytotoxic activity in CD8^+^ T cells. T-bet contributes to B-cell proliferation, surface expression of CD86 and CD21, and antibody IgG2a and IgG2b production by regulating Lyn and Bcl-6. T-bet directly inhibits Tfh differentiation or indirectly suppresses Tfh differentiation by regulating B cells. T-bet inhibits RORγt transcription and then suppresses Th17 cell differentiation and IL-17A production. IFNγ, interferon gamma; JAK, Janus kinase; STAT1, signal transducer and activator of transcription 1; TLR7, toll like receptor 7; IL-21, interleukin-21; MyD88, myeloid differentiation primary response gene 88; USF1, upstream stimulatory factor 1; YY1, Yin Yang 1; Th1, T helper cell 1; Bcl-6, B-cell lymphoma 6; Tfh, follicular T helper cell; RORγt, receptor retinoic acid receptor-related orphan receptor gammat.

T-bet is one of the members of the T-box family of transcription factors (**Figure 1**). It was firstly identified from a Th1 cell cDNA library as a specific transcription factor for Th1 cells. The human T-bet gene (*TBX21*) is located at chromosome 17q21.32. T-bet is expressed in several immune cells such as monocytes and T and B cells. Deficiency of T-bet in CD4^+^ T cells may inhibit secretion of IFNγ, leading to a shift of Th1/Th2 balance to the Th2 pathway. T-bet binds to the promoter of perforin and granzyme B, regulating cytotoxic T lymphocyte activity in CD8^+^ T cells. In a study from southern Chinese SLE patients (without treatment), the percentage of B cells expressing T-bet was increased as compared with that in healthy controls and related to serum levels of anti-dsDNA and anti-C1q antibodies (17). Serum levels of IgM and IgA in SLE patients were also related to T-bet expression in T and B cells (17). In SLE patients of Hong Kong origin (treated with prednisolone, hydroxychloroquine, azathioprine), levels of T-bet were elevated in PBMCs than healthy controls, correlating with serum levels of IFNγ and IL-18 and SLE disease activity (18). Interestingly, SLE patients with a high urinary T-bet expression revealed higher risk of lupus flare and severe flare than SLE patients with a low urinary T-bet expression (19). For rs4794067 in the *TBX21* gene, SLE patients from southern China did not show an association with rs4794067 polymorphism (20). This was inconsistent with another study, where frequencies of rs4794067 T allele and rs17250932 T allele were elevated in northern Chinese SLE patients as compared with controls (21). Frequencies of haplotype CC (rs4794067 (C)+rs17250932(C)) were higher in SLE patients. A study discussed copy number variations (CNVs) in SLE patients from southern Chinese, showing that frequencies of T-bet CNVs in SLE patients were much higher as compared with controls, and expression of T-bet in SLE patients with more than two copies of DNA was higher as compared with patients with two copies of DNA (22). This variation related to T-bet genetics may correlate with different ethnicity, different sample sizes, and detecting methods.

In SLE patients, the TBX21 promoter carrying the rs17250932 C allele showed lower transcriptional activity than patients carrying the rs17250932 T allele, which was further downregulated by overexpressing upstream stimulatory factor 1 (USF-1) (23). USF-1 binds to the rs17250932 C allele more strongly than it binds to the rs17250932 T allele. SLE patients carrying the rs17250932 C allele showed a lower expression of T-bet and IFNγ and an elevated expression of IL-4 in CD4^+^ T cells as compared with patients carrying the rs17250932 T allele (23). Similarly, SLE patients carrying the rs4794067 T allele possessed higher promoter activity when compared with patients carrying the rs4794067 C allele (24). The Rs4794067 C allele strongly binds to the Yin Yang 1 (YY1) transcription factor than the rs4794067 T allele, by which overexpression of YY1 downregulated the TBX21 promoter. SLE patients carrying the rs4794067 C allele showed a lower expression of T-bet and IFNγ when compared with patients carrying the rs4794067 T allele (24). The findings suggested that rs17250932 and rs4794067 polymorphisms inhibit *TBX21* gene expression and Th1 cell-related cytokine generation by binding USF-1 and YY1 to the polymorphisms. BXSB/MpJ-Yaa F1 (Yaa) mice featured autoimmune lupus-like characteristics such as nephritis. Overexpression of T-bet in T cells of the Yaa mice promoted proteinuria and severity of glomerulonephritis and increased the expression of IgG2a and IFNγ, but inhibited the production of IL-4 and IL-5 in serum (25). T-bet^−/−^ B cells had impaired ability to produce IgG2a, IgG2b, and IgG3 (23). Overexpression of T-bet initiates IgG2a switching. In T-bet^−/−^ lupus mice, there were reduction of ANA, anti-dsDNA production, hypergammaglobulinemia, and nephritis development (26). Thus, T-bet may accelerate lupus pathogenesis. In pristane-induced lupus mice, injection of tofacitinib, a Janus kinase (JAK) 1/JAK3 inhibitor, significantly inhibited the expression of suppressor of cytokine signaling (SOCS) 1 but upregulated the expression of SOCS3 (27). SOCS3 transgenic mice showed alleviation of airway obstruction and epithelial loss as compared with those in control mice, along with reduced expression of T-bet in lungs of the mice (28). OT-I CD8^+^ T cells cultured with H2-Kb (SIINFEKL) complex and IL-2 induced ectopic T-bet expression and led to reduced expression of SOCS1 (29). After stimulation of chondrocytes from SOCS3^△/△col2^ mice with IL-6/oncostatin M (OSM), there was persistent activation of STAT3, STAT1, and STAT5 (30). Thus, the SOCS family interacts with signals of the JAK/STAT/T-bet pathway and regulates inflammation or autoimmunity. Interestingly, CD4^+^ T cells had an elevated expression of receptor retinoic acid receptor-related orphan receptor gammat (RORγt) and more IL-17A generation after collage II immunization in T-bet^−/−^ mice (31). T-bet^−/−^ naive CD4^+^ T cells under Th17-polarizing condition showed more Th17 cell differentiation. T-bet^−/−^ mice injected with collage II induced arthritis, along with high expression of RORγt and IL-17A in the arthritic joints. On the contrary, T-bet transgenic mice revealed the suppression of RORγt and IL-17A expression by CD4^+^ T cells under Th17-polarizing conditions and reduced the expression of the IL-6 receptor (32). The findings suggested that T-bet may inhibit Th17 differentiation and related transcription factor and cytokine production.

### 3.2 T-bet is involved in lupus development by regulating B cells

There were more percentages of naive B cells, double-negative (DN) B cells expressing T-bet in SLE patients as compared with controls, and T-bet^+^ DN B cells expressing CD11c (13). A proportion of T-bet^+^ naive B cells were related to IFNγ expression in SLE patients. Patients with a higher proportion of T-bet^+^ naive B cells had a much higher SLE disease activity index (SLEDAI) score, correlating with a higher expression of ANA and anti-dsDNA antibodies and lower disease duration (13). Age-associated B cells (ABCs) are a novel subset of activated B cells, which are involved in protective antiviral response and pathogenesis of autoimmunity. T-bet is considered to be the defining feature of ABC biology. In SLE patients, the frequency of ABCs such as CD11c^+^T-bet^+^CD21^hi^ B cells was related to C3 and C4 expression and estimated glomerular filtration rate (eGFR) (33). After treatment, the frequency of CD11c^+^T-bet^+^CD21^hi^ B cells in complete and partial remission SLE patients was reduced, especially CD11c^+^T-bet^+^CD21^hi^CXCR5^+^ B cells (33). Similarly, the proportion of CD11c^hi^T-bet^+^ B cells was expanded in SLE patients as compared with controls (34). SLE patients with LN showed a higher proportion of CD11c^hi^T-bet^+^ B cells and correlated with the SLEDAI score. Addition of IL-21 promoted CD11c^hi^T-bet^+^ B-cell proliferation and differentiation into Ig-secreting autoreactive plasma cells (34). Administration of the adenosine receptor 2a (A2A) agonist in lupus mice depletes CD11c^+^T-bet^+^ B-cell proliferation, inhibits ANA production in serum, and alleviates kidney pathology and lymphadenopathy (35). Together, T-bet^+^ B cells may be a biomarker for SLE (LN) and IL-21/adenosine-mediated induction of CD11c^hi^T-bet^+^ B cells may regulate lupus development.

In Lyn^−/−^ mice, half of splenic plasma cells (PCs) were differentiated from T-bet^+^ B cells, which were higher than those from WT mice (36). T-bet^+^ B cell-differentiated splenic PCs produced high levels of anti-dsDNA antibody. When inhibiting T-bet^+^ B cells differentiating into PCs or class switching in Lyn^−/−^ mice, there were a reduced number of splenic PCs and a low expression of anti-dsDNA, suggesting that T-bet-expressing B cells contribute to the autoreactive PC pool in Lyn^−/−^ mice (36). This was confirmed in lupus mice with B cells overexpressing T-bet, by which B cells overexpressing T-bet upregulated ANA and anti-dsDNA production and germinal center formation in (NZB×NZW)F1 mice (37). However, inhibiting T-bet expression in B cells impaired the ability to form germinal centers, suppressed kidney damage, and downregulated the activation of total B and T cells in lupus mice (37). Chronic graft-versus-host disease (cGVHD) is a lupus-like syndrome showing much autoantibody generation. Injection of splenocytes from B6(C)-H2-Ab1bm12/KhEgJ mice into WT mice induced cGVHD, evidenced by high percentages of CD11c^+^ B cells and T-bet^+^CD11c^+^ B cells and increased serum levels of anti-chromatin IgG and IgG2a (38). Similarly, a number of T-bet^+^CD11c^+^ B cells were elevated in SLE patients, which was related to serum levels of anti-chromatin IgG. On the contrary, knockdown of T-bet in B cells inhibited cGVHD development and downregulated the production of anti-chromatin IgG (38). CD11c^+^T-bet^+^ ABCs in lupus mice showed a hypermetabolic state with elevated glycolytic capacity, which will be further upregulated by stimulation with IFNγ (39). T-bet repressed Bcl-6 expression and then regulated the gene program of the glycolysis pathway in ABCs. Lupus mice injected with the glycolysis inhibitor inhibited ABC accumulation and ANA and anti-dsDNA generation, indicating that modulation of T-bet-promoted energy metabolism may ameliorate autoimmunity (39). In both B-cell-intrinsic Ship-deficient (ShipΔB) lupus mice and SLE patients, there were high percentages of CD11c^+^T-bet^+^ ABCs and deregulated T follicular helper (Tfh) cells (40). Injection of CD11c^+^T-bet^+^ ABCs into ShipΔB lupus mice will deregulate Tfh cells. Ship^−/−^ B cells stimulated with toll-like receptor 7 (TLR7) will promote CD11c^+^T-bet^+^ ABC differentiation. In ShipΔB lupus mice, ablating MyD88 signaling is able to inhibit CD11c^+^T-bet^+^ ABC differentiation and anti-dsDNA production, normalize Tfh cell differentiation, and rescue antigen-specific germinal center response (40). Therefore, excessive T-bet^+^ B cells promote lupus development. Treg cells have potent immunosuppressive and anti-inflammatory properties. Tfh cells are a subset of CD4^+^ Th cells and help B-cell proliferation and survival. Follicular regulatory T (Tfr) cells are a specific subset of Treg cells and are located in the germinal center (GC). In MRL/lpr mice, the percentage of Tfr cells was lower than that in control mice, whereas the percentage of Tfh cells in MRL/lpr mice was much higher than that in control mice (41). The ratio of Tfr/Tfh cells was lower in lupus mice. However, treatments such as triptolide used for MRL/Lpr mice significantly upregulated the percentage of Tfr cells and ratio of Tfr/Tfh cells (39). In SLE patients, the proportion of Tfr cells was downregulated and the percentage of Tfh cells was higher than that in healthy controls (42). The ratio of Tfr/Tfh cells was related to the expression of anti-dsDNA, IL-17, and SLEDAI in SLE patients. After treatment of SLE patients with low-dose IL-2, the frequency of Tfr cells was increased and the frequency of Tfh cells was reduced, along with restored Tfr/Tfh cell balance (42). In SLE patients treated with low-dose IL-2, there were an increased number of CD4^+^CD25^high^CD127^low^ Treg cells and a reduced number of CD4^+^CXCR5^+^PD-1^+^CCR7l^ow^ Tfh cells, but the number of CXCR3^+^CCR6^-^CCR4^-^CCR7^low^ Th1 cells was not significantly affected (43). In MRL/lpr mice, administration of the artemisinin analog SM934 ameliorated lupus development and suppressed serum levels of IFNγ but increased the expression of Foxp3 in Treg cells (44). Naive CD4^+^ T cells under Th1- and Treg-polarizing conditions in the presence of SM934 showed an elevated percentage of Treg cells and inhibited the development of Th1 cells. In addition, treatment of MRL/lpr mice with the aryl hydrocarbon receptor (AhR) tapinarof strongly upregulated the frequencies of Treg cells and inhibited the proportion of Th1 and Tfh cells (45). It is notable that TBX21^−/−^ B cells had high expressions of CD11b and CD11c after stimulating with combinations of TLR7 agonist, IFNγ, IL-21, anti-CD40 agonist, and anti-IgM (46). B-cell-intrinsic T-bet deletion in lupus mice showed no effect on ABC production, where TBX21^−/−^ ABCs and WT ABCs had identical surface phenotypes, including increased cell size, equivalent IgM and IgD expression, reduced CD21, CD23, and CD138 expression, and upregulated expression of co-stimulatory molecules CD86, MHC II, and CD95. WT ABCs and TBX21^−/−^ ABCs from lupus mice generated comparable expressions of IgM and IgG after TLR7 agonist stimulation, indicating that T-bet is not uniformly required for ABC production (46). This may correlate with several reasons. First, functional ABCs may be produced without B-cell T-bet expression, but the finding did not exclude potential for additional B cell-intrinsic T-bet function in ABC biology and autoimmunity. For example, it is accepted that T-bet is able to upregulate IgG2a and IgG2c class-switch recombination (47) and is necessary for maintenance of IgG2a^+^ and IgG2c^+^ memory B cells (48). The authors found a defect in IgG2c generation from stimulated TBX21^-/-^ ABCs (46). Thus, T-bet^−/−^ B cells may inhibit lupus pathogenesis by downregulating production of IgG2c. Second, the authors showed that T-bet upregulated the percentage of B cells exhibiting ABC surface phenotype after stimulation. In previous studies, Was^−/−^ B cells are hyperresponsive to B-cell receptor (BCR) and TLR stimulation (49, 50). It is hypothesized that different concentrations of TLRs, cytokines, and co-stimulatory signals may lead to TBX21-dependent or -independent ABC production.

## 4 Th1-related inflammatory cytokines

### 4.1 IL-12

IL-12 is a pro-inflammatory cytokine that performs significantly in suppressing virus infection and alleviating the infection symptom. It is highly produced in the host to initiate antiviral immune responses. IL-12 is expressed in innate immune cells, such as monocytes, macrophages, and adaptive immune cells such as T cells. IL-12 binds to receptors on T cells and then controls synthesis of T-bet. In SLE patients, there were increased serum levels of IL-12 as compared with those in healthy controls and were higher in active SLE patients than those in inactive SLE patients (51). Serum levels of IL-12 correlated with the expression of C3. Similarly, in LN patients, there were elevated serum levels of IL-12 than those in non-LN patients, and IL-12 was mainly expressed in glomerular mononuclear cells in class IV and V LN patients, correlating with urinary levels of IL-12 (52). There were a much higher expression of cerebrospinal fluid IL-12 and more atrophy in the medial prefrontal cortex (mPFC) of diffuse neuropsychological SLE (dNPSLE) patients than those in non-dNPSLE patients (53). Sleep disturbance stress-subjected MRL/lpr mice also showed a higher expression of microglial activation-related genes, including *IL-12*. Administration of neutralization of the IL-12 antibody into lupus mice mitigated the stress-induced neuropsychiatric phenotype (53). In MRL/lpr mice, serum levels of IL-12 were higher than those controls, and IL-12 expression in PBMCs and kidney tissues of MRL/lpr mice was increased as well (46). Thus, IL-12 may associate with lupus pathogenesis.

SLE patients’ CD4^+^ T cells under Tfh-polarizing conditions treated with the anti-IL-12 antibody inhibited differentiation of CD4^+^CXCR5^+^PD-1^+^ T cells and CD4^+^CXCR5^+^PD-1^+^Bcl-6^+^ T cells and downregulated IL-21 production (54). TCR-stimulated naive and memory CD4^+^ T cells from MRL/lpr mice showed comparable expression of mitochondrial reactive oxygen species (mROS) (55). Inhibition of mROS reduced IFNγ and CD44 expression. Addition of IL-12 enhanced mROS generation in memory CD4^+^ T cells, suggesting that IL-12 regulates IFNγ and CD44 production via regulating ROS expression (55). *In vivo* studies showed that MRL/lpr mice injected with IL-12 accelerated the pathology of lupus, evidenced by elevated mesangial cell proliferation, mesangial matrix deposition in glomeruli, interstitial cellular infiltration, and higher expression of anti-dsDNA, creatinine, and proteinuria (51). On the contrary, MRL/lpr mice injected with the anti-IL-12 antibody inhibited lupus development, including less mesangial matrix deposition in glomeruli and reduced serum levels of anti-dsDNA, creatinine, and proteinuria (51). In cGVHD mice, injection of the anti-IL-12 antibody also inhibited mesangial cell proliferation and spleen enlargement, downregulated neutrophils in the mesangial area, reduced the renal histopathology score, reduced the serum levels of anti-dsDNA, reduced the number of CD4^+^IFNγ^+^ Th1 cells, CD4^+^IL-17A^+^ Th17 cells, and CD4^+^CXCR5^+^ Tfh cells, and reduced the expression of transcription factors T-bet, Rorγt, Bcl-6, and PU.1, and inflammatory cytokines IFNγ, IL-9, IL-17A, TGF-β1, and IL-21 in spleen (54). Furthermore, a randomized-controlled study discussed the role of ustekinumab (an IL-12 inhibitor) in treatment of active SLE patients, showing that 62% patients who received ustekinumab at week 24 achieved a SLEDAI-2K responder index-4 (SRI-4) response than 33% patients who received placebo (56). Between weeks 0 and 24, 67% patients who received ustekinumab showed at least one adverse event such as infection than 78% patients received placebo, and no deaths, herpes zoster, and malignancies occurred in patients who received ustekinumab (56). In our opinion, IL-12 is a potential and promising therapeutic target for lupus.

### 4.2 IL-18

IL-18 is a pro-inflammatory cytokine, which binds to IL-18 binding protein (IL-18BP) and then regulates downstream signalings, leading to excessive inflammatory responses. IL-18 is expressed in several immune cells such as monocytes and T cells. It is able to activate natural killer (NK), Th1, Th2, and Th17 cells by generating IL-5, IFNγ, IL-4, and IL-17 and promote release of matrix metalloproteinases. The *IL-18* gene is located at chromosome 11 and has seven exons with two distinct promoters (an IFN consensus sequence binding protein and a PU.1 binding site). In SLE patients (unknown treatment), serum IL-18 levels were higher than those in controls (57–61) (**Table 1**). SLE patients with high IL-18 serum levels showed a higher SLEDAI score (57). IL-18 levels in SLE patients were positively related to disease activity, erythrocyte sedimentation rate (ESR), protein/creatinine ratio, anti-dsDNA titers, and endothelial progenitor cell (EPC)/circulating angiogenic cell (CAC) dysfunction and were negatively related to creatinine clearance and C3 expression (58–60, 62, 63). LN patients showed higher serum levels of IL-18 than those in non-LN patients and were higher in class IV and V LN patients than in other classes (59). Serum levels of IL-18 were also elevated in NPSLE patients and positively related to disease activity (64). NPSLE patients with seizure disorders had much higher serum levels of IL-18 than patients with other neuropsychiatric symptoms (64). In PBMCs of SLE patients (unknown treatment), there was a higher expression of IL-18 as compared with that in controls and was positively related to the SLEDAI score and anti-dsDNA titers (65). Interestingly, serum levels of IL-18 in SLE patients treated with glucocorticoids, hydroxychloroquine, and immunosuppressants such as methotrexate, azathioprine, and leflunomide were higher than those in controls (66). LN patients treated with glucocorticoids or glucocorticoids+chloroquine+hydroxychloroquine+azathioprine revealed higher serum levels of IL-18 than LN patients treated with glucocorticoids+cyclophosphamide (67). However, some other studies found that serum levels of IL-18 in the experimental group (traditional Chinese medicine (astragalus+Duhua+Chuanqi+Astragalus membranaceus+Salvia miltiorrhiza+Chinese yam)+western medicine (prednisone+methylprednisolone+cyclophosphamide)) were much lower than those in the control group (western medicine (prednisone+methylprednisolone+cyclophosphamide)) (68). Serum levels of IL-18 in SLE patients after treatment (cyclophosphamide, methylprednisolone, prednisolone, mycophenolate mofetil, azathioprine) were related to poor clinical outcome, relating to SLEDAI score and severity of LN (69). For genetic mutation of IL-18 in SLE, Polish SLE patients carrying the IL-18 rs360719 allele C and CC genotype had a decreased risk of SLE, and the CC genotype was related to LN manifestation (70). However, rs187238 and rs1946518 polymorphisms did not correlate with SLE susceptibility in Polish SLE patients (70). Another study found that rs187238 and rs360719 polymorphisms were related to SLE risk in an Indian population, and the rs360719 CC genotype and allele C were related to LN risk (58). Rs360719 polymorphism was related to SLE patients with a positive anti-nucleosome antibody, and haplotype CC (rs360719 (C)+rs187238 (C)) was positively correlated with serositis and neurologic involvement. Rs1946518 polymorphism did not correlate with SLE risk in an Indian population (58). In Egyptian SLE patients, the frequency of the rs1946518 CC genotype was higher than that in controls and the frequency of genotype CA was lower than that in controls. SLE patients carrying rs1946518 genotype CC showed higher serum levels of IL-18 (62). Based on the findings, it is suggested that levels of IL-18 in SLE patients were abnormal and different kinds of medicine and doses may lead to different expression profiles of IL-18. *IL-18* genetics may associate with SLE.

**Table 1:** Expression profile of IFNγ, IL-18, and TGF-β in lupus.

Cytokine	Year	Author	Country	Sample	Expression status	Treatment	Reference
IFNγ	2013	Lu et al.	China	PBMCs	Increased	Unknown treatment	107^a^
	2015	Kokic et al.	Croatia	Serum	Increased	Prednisolone	103^a^
	2017	Postal et al.	Brazil	Cerebrospinal fluid	Increased	Unknown treatment	108^a^
	2017	Ahn et al.	Korea	PBMCs	Reduced	Glucocorticoids	111^a^
	2018	Wen et al.	China	Serum, urine, renal tissues	Increased	Unknown treatment	106^a^
	2018	Liu et al.	China	PBMCs	Increased	Prednisone, cyclophosphamide, mycophenolate mofetil, methotrexate	104^a^
	2018	Dufour et al.	Canada	PBMCs	Increased	No treatment	105^a^
	2018	Düster et al.	German	Serum	Increased	Unknown treatment	109^b^
	2019	Oke et al.	Sweden	Serum	Increased	Prednisolone	102^a^
	2020	Jiang et al.	China	Serum	Reduced	Cyclophosphamide, prednisolone, traditional Chinese medicine Qing Shen Fang	110^a^
	2021	Raymond et al.	Australia	Serum	Reduced	Prednisone, azathioprine, cyclophosphamide, mycophenolate, hydroxychloroquine, rituximab	112^a^
IL-18	2010	Hu et al.	China	Serum	Increased	Glucocorticoids, chloroquine, hydroxychloroquine, azathioprine	54^a^
	2011	Kahlenberg et al.	Switzerland	Serum	Increased	Unknown treatment	47^a^
	2012	Sahebari et al.	Iran	Serum	Increased	Unknown treatment	48^a^
	2013	Aghdashi et al.	Iran	Serum	Increased	Unknown treatment	50^a^
	2014	Fouad et al.	Egypt	Serum	Increased	Unknown treatment	49^a^
	2016	Jafari-Nakhjavani et al.	Iran	Serum	Increased	Unknown treatment	46^a^
	2016	Wu et al.	Taiwan	Serum	Reduced	Cyclophosphamide, methylprednisolone, prednisolone, mycophenolate mofetil, azathioprine	56^a^
	2018	Mende et al.	Australia	Serum	Increased	Glucocorticoids, hydroxychloroquine, methotrexate, azathioprine, leflunomide	53^a^
	2019	Umare et al.	India	Serum	Increased	Unknown treatment	45^a^
	2020	Miteva et al.	Bulgaria	PBMCs	Increased	Unknown treatment	52^a^
	2021	Rezaieyazdi et al.	Iran	Serum	Increased	Unknown treatment	44^a^
	2022	Liang et al.	China	Serum	Increased	Unknown treatment	51^a^
	2023	Ye et al.	China	Serum	Reduced	Traditional Chinese medicine astragalus, Dahua, Chuanqi, Astragalus membranaceus, Salvia miltiorrhiza, Chinese yam and prednisone, methylprednisolone, cyclophosphamide	55^a^
TGF-β	2010	Becker-merok et al.	Norway	Serum	Reduced	Prednisolone	82^a^
	2012	Jin et al.	Sweden	Serum	Reduced	Glucocorticoids	83^a^
	2012	Ou et al.	USA	Serum	Increased	Unknown treatment	96^a^
	2014	Sayed et al.	Egypt	Serum	Reduced	Unknown treatment	85^a^
	2017	Yuan et al.	China	PBMCs	Increased	Unknown treatment	88^a^
	2018	Menyawi et al.	Egypt	Serum	Reduced	Unknown treatment	84^a^
	2019	Rashad et al.	Egypt	Serum	Reduced	Unknown treatment	86^a^
	2019	Paradowska-Gorycka et al.	Poland	Serum	Increased	Unknown treatment	87^a^
	2021	Stadtlober et al.	Brazil	Serum	Unknown	Prednisone, mycophenolate	94^a^
	2022	Yuliasih et al.	Indonesia	Serum	Increased	Unknown treatment	91^a^
	2023	Gómez-Bernal et al.	Spain	Serum	Unknown	Prednisone, hydroxychloroquine, methotrexate, mycophenolate mofetil, azathioprine, rituximab, belimumab	89^a^
	2023	Gómez-Bernal et al.	Spain	Serum	Unknown	Prednisone, hydroxychloroquine, methotrexate, mycophenolate mofetil, azathioprine, rituximab, belimumab	90^a^

a Human.

b Mouse models.

PBMCs, peripheral blood mononuclear cells.

Since serum levels of IL-18 correlated with EPC/CAC dysfunction, stimulating EPC/CAC from SLE patients with IL-18 suppressed endothelial differentiation and neutralization of IL-18 in EPC/CAC-upregulated differentiation of mature endothelial cells, indicating a deleterious role of IL-18 in vascular repair (60). In (NZB×NZW)F1 mice, the blood–brain barrier (BBB) was intact and the mice showed hippocampus-related behavioral deficits recapitulating dNPSLE, evidenced by disrupted hippocampal neurogenesis with hiNSCs showing elevated proliferation, apoptosis, and decreased differentiation (71). When hiNSCs were stimulated with IL-18, there was induced apoptosis of the cells, suggesting that microglial activation in the presence of intact BBB and disruption of hippocampal neurogenesis by IL-18 mediate dNPSLE (71). Moreover, IL-18^−/−^ MRL/lpr mice showed reduced signs of renal pathogenesis, such as less sclerotic changes of the mesangium, protein casts in distal tubuli, and lobular accentuation of glomerulopathy (72). Together, IL-18 may contribute to lupus development.

### 4.3 TNFα

**Figure 2:**
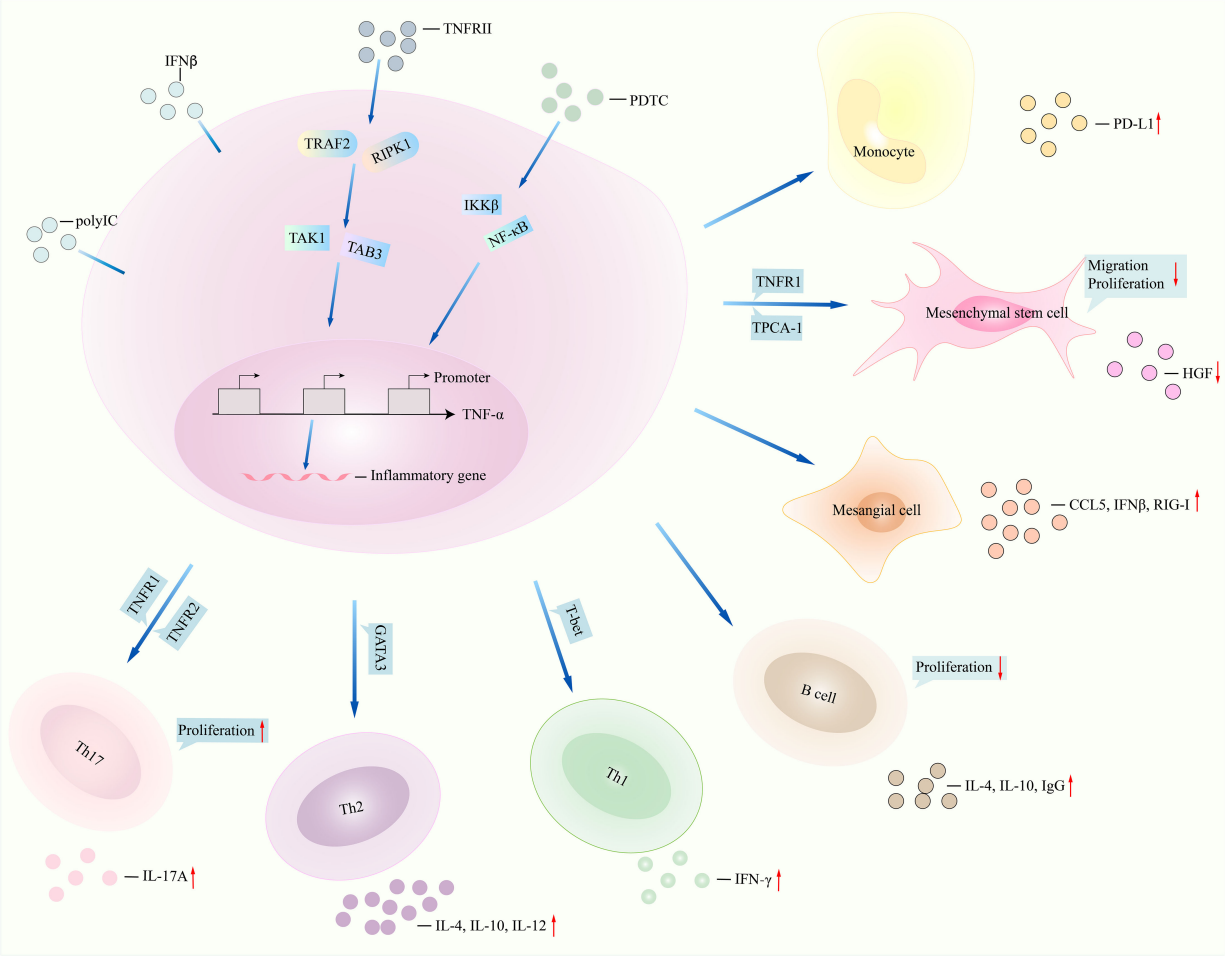
TNFα signalings in lupus. NF-κB inhibitor pyrrolidine dithiocarbamate (PDTC) regulates downstream signalings such as IKKβ and NF-κB and then inhibits function of TNFα, for instance, less production of inflammatory genes CD68 and IL-6. TNFRII interacts with TRAF2 and RIPK1 and then regulates the expression of TNFα by interacting with TAK1 and TAB3. Monocytes stimulated with TNFα promoted PD-L1 expression. Migration, proliferation of mesenchymal stem cells, and production of HGF were reduced after TNFα interacted with TNFRII and TPCA-1. TNFα induced the production of CCL5, IFNβ, and retinoic acid-inducible gene-I (RIG-I) in mesangial cells under polyinosinic-polycytidylic acid (poly IC) stimulation. Inhibition of TNFα in T cells and B cells leads to less IL-4 and IL-10 generation. TNFα regulates TNFR1 and TNFR2 and then promotes the expression of IL-17 and Th17 cell proliferation. NF-κB, nuclear factor kappa B; IKKβ, inhibitor of nuclear factor kappa B kinase subunit beta; HGF, hepatocyte growth factor (HGF); TNFα, tumor necrosis factor-alpha; IL-6, interleukin-6; TNFRII, tumor necrosis factor receptor II; TRAF2, tumor necrosis factor receptor-associated factor 2; RIPK1, receptor-interacting protein kinase 1; TAK1, transforming growth factor beta-activated kinase 1; TAB3, TAK1-binding protein 3; PD-L1, programmed cell death ligand 1; TPCA-1, 2-[(aminocarbonyl)amino]-5-(4-fluorophenyl)-3-thiophenecarboxamide. CCL5, C-C motif chemokine ligand 5; IFNβ, interferon beta. TNFR1, TNFα receptor 1.

TNFα is a pro-inflammatory cytokine generated by some immune cells, for instance, monocytes, macrophages, neutrophils, T cells, and B cells (**Figure 2**). It is primarily generated as a trans-membrane protein with 17 kDa. TNFα performs different biological functions, by which it not only can initiate host defense against infection but also is involved in toxicity and inflammatory responses. SLE patients showed elevated plasma levels of TNFα as compared with those in healthy controls, correlating with SLEDAI score, high plasma levels of IFNα, and programmed death ligand-1 (PD-L1) (73–77). Bone marrow (BM) from SLE patients revealed a striking death of niche and hematopoietic cells were related to high expression of TNFɑ (78). LN patients reported higher plasma levels of TNFα as compared with non-LN patients (74). Interestingly, in SLE patients with depressive symptoms and anxiety symptoms, serum and cerebrospinal fluid (CSF) levels of TNFα were higher than those in patients without depressive symptoms and anxiety symptoms, correlating with poorer health-related quality of life (HRQoL) (79–81). When discussing serum levels of TNFα in SLE patients, unaffected family members, and healthy controls, SLE patients showed the highest TNFα levels, and unaffected first-degree relatives had higher serum levels of TNFα than the healthy controls, suggesting that TNFα expression may correlate with heritable factors (82). Regarding TNFα genetic mutation, the rs1800629 A allele was positively related to SLE risk in Indian, Chinese, and Bulgarian populations (73, 75, 83, 84). The rs1800629 A allele was negatively related to SLE risk in a Polish population (85), whereas rs1800629 was not related to SLE risk in a Mexican population (86). Rs1800629 GA and AA genotypes were associated with SLE patients with LN and hematological manifestations and were positively related to SLE patients with anti-dsDNA(+) and anti-Sm(+) antibodies in an Indian population (85, 87). For rs361525, GA genotype and A allele were associated with SLE risk in Indian and Mexican populations (83, 84) whereas rs361525 was not related to SLE risk in a Polish population (83). Haplotype CAGA (rs1799964 (C)+rs1800750 (A)+rs1800629 (G)+rs361525 (A)) was positively related to SLE susceptibility, and polymorphisms rs1800750 and rs1799964 correlated with SLE risk in a Mexican population as well (84). It is notable that Indian SLE patients carrying the TNFα rs361525 GG genotype and the rs1800629 GG genotype were related to lower plasma levels of TNFα as compared with patients carrying the TNFα rs361525 GA or AA genotype and the rs1800629 GA or AA genotype, respectively (73). On the contrary, Polish SLE patients carrying the rs361525 GG genotype and rs1800610 CC genotype had higher plasma levels of TNFα as compared with patients carrying the rs361525 GA or AA genotype and the rs1800610 CT or TT genotype, respectively (85). Collectively, SLE showed that aberrant expression of TNFα and gene polymorphisms of TNFα may relate to SLE risk. However, different results of TNFα gene polymorphisms in SLE may correlate with ethnicity, sample sizes, and detecting methods. Thus, larger samples and multicenter studies are needed in the further to confirm the above findings.

TNFα regulates inflammation and autoimmunity development, including SLE. Monocytes from SLE patients treated with TNFα significantly produced PD-L1 expression, whereas monocytes treated with a TNFα-neutralizing antibody will block production of PD-L1 (78). Migration rate, proliferation of SLE bone marrow-derived mesenchymal stem cells (BMSCs), and hepatocyte growth factor (HGF) expression were lower than those in healthy controls, which was further suppressed by addition of SLE serum or TNFα stimulation (85) but was upregulated by stimulating with IKKβ inhibitor 2-[(aminocarbonyl)amino]-5-(4-fluorophenyl)-3-thiophenecarboxamide (TPCA-1). Expression of TNF receptor I (TNFRI) and phosphorylated-I kappa B kinase beta (pIKKβ) was elevated in SLE BMSCs. Inhibition of TNFRI converted TNFα-induced impaired the migration and proliferation ability of SLE BMSCs. HGF expression in SLE BMSCs could be downregulated by culturing with TPCA-1 or TNFRI-neutralizing antibody. Expression of pIKKβ was increased after stimulating SLE BMSCs with TNFα. In lupus mice, there was impaired *in vivo* homing capacity of SLE BMSCs upon TNFα stimulation, evidenced by massively trapped BMSCs within the lungs. However, addition of a TNFRI-neutralizing antibody to TNFα-treated lupus mice induced less BMSCs within the lungs but more BMSCs homing to the lymph node and spleen. The finding indicated that TNFα-induced impaired migration of SLE BMSCs was mediated by activating IKKβ and then inhibiting HGF (87). Mesangial cells from LN patients cultured with polyinosinic–polycytidylic acid (poly IC) will upregulate the expression of CCL5, IFNβ, and retinoic acid-inducible gene-I (RIG-I), and addition of TNFα further enhanced the poly IC-induced expression of CCL5, IFNβ, and RIG-I (88). Knockout of IFNβ in (poly IC) and TNFα-treated mesangial cells suppressed the production of CCL5 and RIG-I. Similarly, knockout of RIG-I in (poly IC) and TNFα-treated mesangial cells suppressed the production of CCL5, indicating that TNFα results in activation of the TLR3/IFNβ/RIG-I/CCL5 axis (88). Moreover, there was increased expression of TNFα and pp65 in the cerebral cortex and hippocampus, long immobility period of MRL/lpr mice, and low expression of pIκBα in the hippocampus and total and center track lengths of lupus mice (79). The MRL/lpr mice showed depression behavior. Upon stimulating with TNFα, proliferation of microglial cell line (BV2) cells was upregulated, showing round-shaped microglia and elevated expression of IL-6, CD68, and NF-κB activity. By contrast, BV2 cells treated with TNF-α in the presence of NF-κB inhibitor pyrrolidine dithiocarbamate (PDTC) suppressed the expression of CD68 and IL-6 and activity of NF-κB, implying that TNFα controls microglial activation by NF-κB signaling in lupus with depression (79). Injection of TNFα antisense oligonucleotides (ASO) into MRL/lpr mice strongly inhibited the expression of TNFα, IFNγ, IL-4, IL-10, and IL-12 and the number of DN T cells and total B cells in the spleen and lymph node, prolonged the life span of MRL/lpr mice, and reduced proteinuria, mesangioproliferative glomerulonephritis, IgG deposition, and anti-dsDNA titers (89). Injection of (NZB×NZW)F1 mice with the TNF receptor type II (TNFRII) antibody stabilized nephritis, prolonged the survival of the mice, and inhibited glomerular IgG deposition, along with reduced renal expression of CCL5, CCL2, and CCL9, endothelial cell activation, interstitial macrophage accumulation, and tubular damage (90). Moreover, (NZB×NZW)F1 mice that received TNFα antagonist etanercept treatment showed reduced mean arterial pressure, albuminuria, glomerulosclerosis, and downregulated percentage of renal cortex CD68^+^ cells, renal cortex NADPH oxidase activity, and renal cortex NF-κB expression (91). Similarly, FcγRIIb^−/−^ lupus mice treated with etanercept upregulated cancellous bone volume and cortical thickness and downregulated expression of osteoblast marker genes (*OSX*, *Collagen type I α 1*) and serum levels of TNFα, IFNγ, IL-6, and IL-17A (92). In FcγRIIb^−/−^ lupus mice, TNFα expression in macrophages was elevated (93). FcγRIIb^−/−^ mice treated with the TNFα blocker (murine p75TNFR : Fc) markedly downregulated proteinuria, tubulo-interstitial damage, serum levels of anti-dsDNA, renal expression of heparin-binding EGF, profibrotic factor connective tissue growth factor, collagen type I α1, and fibronectin, whereas it improved glomerular scores (93). The above findings were demonstrated in TNFα^−/−^ mice, where TNFα^−/−^ mice injected with pristane reported absent hypocellularity, less extensive extramedullary hematopoiesis in livers, and suppressed expression of CXCL12, anti-Sm, and anti-RNP antibodies and percentage of CD19^+^CD138^in^ plasmablasts (78, 94). Thus, TNFα contributes to lupus development *in vivo* and *in vitro*. Moreover, Th17 cells stimulated with TNFα promoted the expansion of the Th17 cells, and blockade of TNFR1 (TNFα receptor (TNFR)1) or TNFR2 on Th17 cells showed that there was augmented expression of IL-17 (95), suggesting that TNFα promoted Th17 cell proliferation by regulating TNFR1 and TNFR2. RA patients who received TNFα inhibitors showed improved clinical condition, evidenced by decreased proportion of Th17 cells and reduced serum levels of IL-17 (96). Injection of a fusion protein targeting TNFα into collagen-induced arthritis mice showed reduced ratio of Th17 cells in the spleens (97). Thus, TNFα positively regulates Th17 cell function.

### 4.4 TGF-β

TGF-β is a pleotropic inflammatory cytokine that is involved in a variety of biological processes. This group of cytokine includes three isoforms, namely, TGF-β1, TGF-β2, and TGF-β3. The three members of the TGF-β family have 70%–82% amino acid homology. TGF-β1 is a secreted protein, which may regulate immune cell growth, proliferation, and differentiation. TGF-β2 may control the function of cardiovascular, pulmonary, and urogenital systems. TGF-β3 is able to directly bind to the TGF-β type II receptor (TGF-βR2), showing isoform-specific biological activity. SLE patients from a Norwegian population treated with steroid or cytotoxic drug showed lower serum levels of TGF-β1 than in controls, and levels of TGF-β1 were related to number of CD4^+^, CD8^+^ T cells, and NK cells (98) (**Table 1**). Serum levels of TGF-β1 were also decreased in Swedish SLE patients after treatment with glucocorticosteroid, correlating with the number of white blood cells, platelet counts, and eGFR (99). TGF-β1 levels were lower in SLE patients with high disease activity and severe organ damage (99). Some other studies showed that serum levels of TGF-β1 were lower in Egyptian SLE patients (unknown treatment) than those in controls, and LN patients (unknown treatment) revealed lower serum levels of TGF-β1 than in non-LN patients (100–102). Reduced serum levels of TGF-β1 were related to disease duration, patients with discoid rash, fever, arthritis, proteinuria, hematuria, and SLEDAI score, and serum levels of cholesterol and triglyceride (100–102). On the contrary, there were higher serum levels of TGF-β1 in Polish SLE patients (unknown treatment) than in healthy controls (103), and northern Chinese SLE patients (unknown treatment) had an increased expression of TGF-β1 in PBMCs than in controls (104). Moreover, serum levels of TGF-β1 in Spanish SLE patients treated with prednisone, hydroxychloroquine, methotrexate, mycophenolate mofetil, azathioprine, rituximab, and belimumab were related to high levels of LDL/HDL ratio, Katz severity index, atherogenic index, carotid plaque, and ocular and cardiovascular manifestations (105, 106). Indonesian SLE patients (unknown treatment) with systemic lupus activity measurement (SLAM) score >30 revealed higher serum levels of TGF-β1 and IL-6 and lower serum levels of C3 and C4 (107). Patients with high serum creatinine levels showed higher serum levels of TGF-β1 than patients with low serum creatinine levels (107). In SLE patients (unknown treatment) from North America, serum levels of TGF-β1 were negatively related to PD-L1 expression. Therefore, the expression of TGF-β1 was abnormal in SLE patients; however, whether this cytokine is upregulated in SLE patients or reduced in SLE patients needs further studies with multiple sample sizes and consistent treatment. For *TGF-β1* gene polymorphisms with SLE, the TGF-β1 rs1800469 C allele was negatively related to SLE risk in a UK population and the rs1800469 C allele was related to SLE patients with neurological disease (108). *TGF-β1* gene variants (rs1800469, rs1800470) did not associate with SLE risk in an Iranian population (108). In Brazilian SLE patients, the TGF-β1 rs1800470 CC genotype was positively related to SLE susceptibility and was related to reduced serum levels of C4 (109). SLE patients with TGF-β1 rs1800470 TC and CC genotypes showed lower serum levels of TGF-β1 as compared with that in patients carrying the TT genotype. The TGF-β1 rs1800469 TT genotype was related to decreased serum levels of C4 (109). In Polish SLE patients, there were elevated frequencies of the TT genotype and T allele of TGF-β1 rs1800469 polymorphism in SLE patients, and there were more frequencies of the TGF-β1 rs1800470 C allele in SLE patients. Rs1800469 polymorphism correlated with the expression of C-reactive protein (CRP), erythrocyte sedimentation rate, hemoglobin, and titers of the anti-Sm antibody. SLE patients carrying the TGF-β1 rs1800469 TT genotype showed higher serum levels of TGF-β1 (103). Frequencies of genotypes and allele of rs1800470 were not different between Egypt SLE patients and healthy controls, or between LN patients and non-LN patients. Serum levels of TGF-β1 were lower in SLE patients carrying the TT genotype than in patients carrying CC+TC genotypes (101). Considering these differences for rs1800469 and rs1800470, it is hypothesized that different ethnicity, sample sizes, and genotyping methods may be different for the studies.

Whole blood cells from SLE patients stimulated with TGF-β1 upregulated the expression of platelet-derived growth factor B (PDGF-B), whereas addition of the TGF-β1 antagonist or neutralizing anti-TGF-β1 antibody significantly suppressed production of PDGF-B (107). PBMCs from SLE patients stimulated with IL-2 promoted production of IFNγ and increased proliferation of PBMCs; however, administration of TGF-β1 in IL-2-treated PBMCs suppressed IFNγ production and proliferation of PBMCs and downregulated Smad family member 7 (Smad7) expression in PBMCs (110). SLE monocytes treated with TGF-β1 inhibited PD-L1 expression (77). Interestingly, B cells from SLE patients cultured with TGF-β3 showed reduced B-cell survival, proliferation, and differentiation into plasmablasts and IgG, IgA, and IgM production and decreased the expression of IFN regulatory factor 4 (IRF4), B lymphocyte-induced maturation protein-1 (Blimp-1), X-box-binding protein 1 (XBP1), Smad1/5, and Syk (111). T peripheral helper (Tph)-like cells in SLE patients such as PD-1^hi^MAF^+^ cells, IL-21^+^IL-10^+^ cells, and PD-1^hi^CX3CR1^+^ cells stimulated with TGF-β3 showed increased proliferation of the cells and elevated expression of PDCD-1, SOX4, and CXCL13 (112). CXCR5^-^PD-1^hi^ Tph cells cocultured with class-switch memory B cells in the presence of TGF-β3 revealed much class-switch memory B cells differentiated into plasmocytes and high expression of IgG and IgM, suggesting that TGF-β3 regulates differentiation and function of Tph-like cells in lupus (112). Injection of the poly(lactic-co-glycolic acid) nanoparticle encapsulating TGF-β into lupus mice induced expansion of CD4^+^Foxp3^+^ Treg cells and CD8^+^Foxp3^+^ Treg cells and suppression of anti-dsDNA antibody and reduced renal disease (113). Adoptive transfer of TGF-β1-treated CD8^+^CD103^+^ Treg cells to cGVHD mice significantly reduced anti-dsDNA antibody and proteinuria, downregulated renal pathological lesions, and lowered the renal deposition of IgG (114). Coculturing TGF-β-treated Treg cells with B cells from (NZB×NZW)F1 mice strongly inhibited IgG production (115). After adoptively transferring TGF-β-treated Treg cells into lupus mice without endogenous T cells, there were decreased serum levels of anti-dsDNA, proteinuria, and lower percentage of CD138^+^ plasma cells (116). Together, TGF-β and its subfamily may have different roles in lupus-related inflammatory components production and lupus development.

### 4.5 IFNγ

IFNγ is primarily generated by immune cells, for example, T cells, macrophages, and NK cells. It is able to regulate both innate and acquired immune responses such as antigen presentation and phagocytosis. The IFNγ gene is located at chromosome 12q24. IFNγ is capable of playing roles in antiproliferative, antiviral, and immunomodulatory activities. SLE patients treated with prednisolone showed higher serum levels of IFNγ than healthy controls, which were higher in SLE patients with fever, rash, arthritis, and LN (116, 117) (**Table 1**). Similarly, SLE patients treated with prednisone, cyclophosphamide, mycophenolate mofetil, and methotrexate revealed elevated expression of *IFNγ* and *TBX21* in PBMCs and increased percentage of IFNγ-producing cells, which were related to expression of anti-dsDNA and SLEDAI score (118). Compared with SLE patients with low expression of IFNγ, SLE patients with high expression of IFNγ reported a higher SLEDAI score (118). In untreated SLE patients’ PBMCs, the expression of IFNγ response genes (*IFNGR1*, *IFGNR2*, *ITGAM*, *S100A8*, *S100A9*, *STAT1*) was higher than that in controls (119). In SLE patients treated with corticosteroid and hydroxychloroquine, the expression of IFNγ response genes was similar to that of controls. With increasing SLEDAI score and clinical deterioration of SLE patients, there was an elevated expression of IFNγ response genes. By contrast, in SLE patients reporting clinical improvement, there was a reduced SLEDAI score and expression of IFNγ response genes (120). Several other studies also discussed the expression profile of IFNγ in SLE patients or lupus mice (unknown treatment), showing that serum, urine, and CSF levels of IFNγ and the percentage of IFNγ^+^γδ^+^ T cells in SLE patients were increased than in controls (120–122). LN patients displayed higher serum levels of IFNγ than in non-LN patients. The expression of IFNγ in renal tissues from LN patients was higher than in non-LN patients (120). NPSLE patients revealed increased CSF IFNγ levels and cerebral volume reduction compared with controls (122). Cerebral volume reduction was related to CSF IFNγ levels in NPSLE patients (122). Serum levels of IFNγ in MRL/lpr mice were increased compared to control mice (123). However, SLE patients treated with cyclophosphamide, prednisolone, and traditional Chinese medicine Qing Shen Fang showed lower serum levels of IFNγ, ESR, and SLEDAI score than patients treated with cyclophosphamide and prednisolone (124). The expression of IFNγ in phytohemagglutinin-stimulated PBMCs from active SLE patients treated with the TNFα inhibitor was lower than that in inactive SLE patients and was correlated with the SLEDAI score (125). In smoking SLE patients, there were prevalent malar rash, mucosal ulcers, arthritis, and Raynaud’s phenomenon, elevated usage of prednisone and hydroxychloroquine, and reduced serum levels of IFNγ (126). Thus, the expression of IFNγ may be a potential biomarker for SLE. However, current evidence cannot conclude the exact expression profile of IFNγ in SLE, where different treatments and different sources have revealed different expressions of IFNγ. For genetic polymorphism of the *IFNγ* gene and SLE risk, available evidence focuses on rs2430561. In a southern Chinese Han population, IFNγ rs2430561 polymorphism was associated with increased risk of SLE, where frequencies of TA and TA+AA genotypes were elevated in SLE patients (122). rs2430561 was related to SLE patients complicated with LN, proteinuria, and anti-dsDNA/anti-Sm antibodies (127). In Egyptian SLE cases, there were higher frequencies of AT+TT genotypes as compared with that in controls (128). Similarly, Brazilian SLE patients carrying the AT genotype revealed a higher risk of SLE (129). However, in Iranian SLE patients, rs2430561 was not related to SLE risk (130). These inconsistencies may relate to different ethnicities and sample sizes.

STAT1 expression was increased in PBMCs from SLE patients, correlating with SLEDAI score and expression of CD95, HLA-DR, and MHC class I (131). The expression of IFNγ response gene *IP-10* was elevated in SLE patients’ monocytes, and stimulation of SLE PBMCs with IFNγ significantly induced STAT1 phosphorylation, suggesting that IFNγ may be involved in SLE pathogenesis via regulating STAT1 signaling (131). Similarly, SLE PBMCs stimulated with IFNγ inhibited the expression of vitamin D receptor (VDR) and microRNA-125b (miR-125b), and knockdown IFNγ expression in THP-1 cells revealed a high expression of VDR and miR-125b (132). After inhibiting the expression of miR-125b in THP-1 cells, the expression of VDR was downregulated, indicating that IFNγ inhibited miR-125b and then suppressed VDR expression (132). When PBMCs from SLE patients were cocultured with umbilical cord-derived mesenchymal stem cells (UC MSCs), UC MSC proliferation was elevated, the expression of vascular endothelial growth factor (VEGF) and CXCL12 was increased, and Akt/IκB/STAT5 signaling pathways were activated (133). IFNγ-stimulated UC MSCs showed elevation of indoleamine 2,3-dioxygenase 1 (IDO1), IDO2, cytochrome oxidase c subunit 1 (COX-1), and IL-6. On the contrary, addition of anti-IFNγ antibody to coculture of PBMCs and UC MSCs will suppress the expression of IDO1 and IDO2 (134). Culturing CD11b^+^ cells with serum from SLE patients significantly activated CD11b^+^ cells, and addition of IFNγ further enhanced the activation of CD11b^+^ cells (134). SLE patients’ lesional skin showed low expression of barrier genes (*loricrin*, *claudin 1*, *transglutaminase 5*) (135). After keratinocytes were exposed to IFNγ, there was a high expression of intercellular adhesion molecule 1 (ICAM1), low expression of genes involved in formation of cornified envelope (*filaggrin*, *loricrin*), tight-junction proteins (*desmoglein 1*, *filaggrin family member 2*), and genes related to extracellular matrix formation (*fibrinogen γ*, *fibronectin*). Keratinocytes infected with *Staphylococcus aureus* and then stimulated with IFNγ upregulated *Staphylococcus aureus* adherence and repressed *Staphylococcus aureus* invasion, implying that *Staphylococcus aureus* colonization is increased on lupus skin lesions and is promoted by IFNγ-mediated barrier disruption (135). In lupus mice, stimulating bone marrow-derived CD11b^+^ cells with IFNγ resulted in elevation of FcγR1 and FcγR3 (134). When incubating CD11b^+^ cells with serum from FcγR2b^−/−^ lupus mice, there was high phosphorylation expression of Syk and ROS and activation of CD11b^+^ cells. Addition of IFNγ further upregulated ROS activity in CD11b^+^ cells. However, stimulating CD11b^+^ cells with piceatannol blunted ROS production, indicating that IFNγ licenses CD11b^+^ cells in lupus by regulating Syk signaling (134). It is known that GATA-3^+^IL-13- and IL-5-producing group 2 innate lymphoid cells (ILC2s) are able to promote tissue repair in barrier organs. In MRL/lpr mice, there were a limited number of ILC2s in inflamed renal tissue. Lin^−^CD127^+^Sca-1^+^CD25^+^ ILC2s from MRL/lpr mice cultured with IL-33 led to activation of ILC2s, increased ILC2 numbers, and more production of IL-5 and IL-13 (123). Interestingly, addition of IFNγ to IL-33-treated ILC2s completely suppressed the effects of IL-33, suggesting that IFNγ suppresses IL-33-mediated response of ILC2s (123). Together, all the findings demonstrated that IFNγ promotes inflammatory components production and different immune cell and non-immune cell dysfunction.

When discussing the role of IFNγ in lupus development or potential of targeting IFNγ in lupus, both lupus mouse models and SLE patients with *in vivo* studies have implied a significant association of IFNγ and lupus. In lupus-prone Roquin^san/san^ mice, reduced IFNγ decay led to much IFNγ signaling in T cells, accumulation of Tfh cells, spontaneous germinal center formation, ANA production, and severe nephritis (136). IFNγR^−/−^ Roquin^san/san^ mice showed prevented lupus development, evidenced by low number of Tfh cells and reduced ANA production, whereas IFNγR^+/+^ Roquin^san/san^ mice upregulated the Bcl-6 expression in Tfh cells (136). Similarly, Deborah et al. generated a mouse model with a 162-nt AU-rich element (ARE) region deletion in the 3′UTR of the *IFNγ* gene that led to high serum levels of IFNγ (137). Mice with homozygous ARE deletion (ARE-Del^−/−^) showed a high number of pDCs, absence of marginal zone B cells, and marginal zone macrophages in bone marrow and spleen. ARE-Del^+/−^ mice had marginal zone B cells and marginal zone macrophages and did not develop lupus. In ARE-Del^−/−^ mice, there were elevated serum levels of IgG2c, ANA, and anti-dsDNA antibodies, decreased serum levels of IgG1 and IgG3, thickened mesangium and complement deposition over the glomerular mesangium, and no evidence of capillaries (137). Furthermore, SLE patients treated with anti-IFNγ monoclonal antibody AMG 811 showed reduced serum levels of IP-10 and less adverse events as compared with those in SLE patients treated with placebo (138, 139). Thus, IFNγ excess may result in Tfh cell accumulation, mutation of IFNγ may promote lupus development, and targeting IFNγ may be a potential for SLE.

### 4.6 IL-2

IL-2 was initially considered as a potent T-cell growth factor and then identified as a cytokine in immune tolerance and immune regulation. It can be generated by T cells, DCs, and monocytes. IL-2 is important in immune cell survival, expansion, and immunity against infection. Serum levels of IL-2 were higher in SLE patients (unknown treatment) from southern China than in controls (case–control study) and were related to the SLEDAI score (140). Another study showed that serum levels of IL-2 in SLE patients from southern China were reduced as compared with those in controls, by which serum levels of IL-2 in newly diagnosed SLE patients (without treatment) were lower than that in treated SLE patients (case–control study) (141). Serum levels of IL-2 in SLE patients were negatively related to the SLEDAI score. By contrast, serum levels of soluble IL-2Rɑ (sIL-2Rα) in SLE patients were higher than in controls (case–control study), and levels of sIL-2Rα in newly diagnosed SLE patients were higher than in treated SLE patients. The expression of sIL-2Rα in SLE patients was positively related to the SLEDAI score and negatively related to serum levels of IL-2 (141). For IL-2 gene polymorphism, the IL-2 rs2069762 T allele and GT genotype were negatively related to SLE risk from an Iranian population and haplotype GT [rs2069762 (G)+rs2069763 (T)]= was positively related to SLE risk (case–control study) (130). The findings indicated that the expression of IL-2 was aberrant in SLE patients and IL-2 genetic mutation may correlate with SLE pathogenesis.

To date, much more studies have pointed the role of IL-2 in regulating inflammatory component production either from human beings or from animal models, and recent studies are comprehensively discussing the potential for targeting IL-2 in lupus. In this part, we mainly discussed the significant role of targeting IL-2 in SLE patients or animal models. Hopefully reviewing the updated advancement of IL-2 and SLE will more clearly understand the current focus of IL-2 on SLE. IL-2 signals by high-affinity trimeric IL-2R in a non-covalent manner or an intermediate-affinity dimeric IL-2R. Zhang et al. incorporated fluorosulfate-L-tyrosine (FSY) into IL-2 to produce an IL-2 variant that binds to IL-2Rα, and FSY substitution at the L72 site of IL-2 (L72-FSY) activates Tregs (142). Culturing SLE patients’ PBMCs with L72-FSY upregulated the number of Treg cells and the expression of CD25 and Foxp3 (142). SLE PBMCs stimulated with exogenous sIL-2Rα downregulated the number of Treg cells (131). SLE PBMCs stimulated with IL-2 upregulated the expression of CD25, Foxp3, and Bcl-2 in Treg cells and the frequency of CD25^hi^ Treg cells and promoted proliferation of CD3^−^CD56^+^ NK cells and expression of Bcl-2 in NK cells and in CD3^+^CD56^+^ natural killer T (NKT) cells (143). There was a low percentage of IL-2-producing CD4^+^ T cells in SLE patients, and CD4^+^ T cells from SLE patients stimulated with IL-2 reduced the percentage of pSTAT5^+^CD4^+^ T cells and inhibited the proliferation of CD4^+^ T cells and expression of phosphorylation of JAK3 (144). CD4^+^ T cells under Treg-polarizing conditions in the presence of anti-IL-2 antibody inhibited Treg cell differentiation and downregulated STAT5 phosphorylation expression (145). CD4^+^ and CD8^+^ T cells stimulated with IL-2 promoted the expression of IL-5, IL-13, and IFNγ whereas neutralization of IL-2 reduced the phosphorylation expression of STAT6 and GATA-3 in CD8^+^ T cells (145). In SLE patients, there was a high proportion of CD4^+^CXCR5^+^Foxp3^−^PD-1^hi^ Tfh cells and a low proportion of CD4^+^CXCR5^+^CD45RA^-^Foxp3^hi^ Tfr cells (146). Stimulating Tfh cells with IL-2 converted Tfh cells to CXCR5^+^Bcl-6^+^Foxp3^hi^pSTAT3^+^pSTAT5^+^ Tfr cells. Addition of IL-2 to coculture of Tfh and B cells will inhibit the proportion of CD38^+^CD27^hi^ plasmablasts (146). For lupus mice, coculturing UC MSCs with splenocytes upregulated the expression of IL-2 and the proportion of Treg cells. Interestingly, addition of an IL-2-neutralizing antibody downregulated Treg cell differentiation (147). There was a high proportion of splenic IL-17^+^ γδ T cells in MRL/lpr mice, and culturing IL-17^+^ γδ T cells with IL-2 inhibited IL-17^+^ γδ T cell survival and downregulated RORγt expression (148). Addition of the STAT5 inhibitor to IL-2-stimulated IL-17^+^ γδ T cells further suppressed RORγt expression. All these indicated that IL-2 regulates inflammation occurrence by regulating downstream signalings.

Numerous studies have recently discussed IL-2 in treating SLE patients (**Table 2**). A total of eight studies evaluated the effect of low-dose human IL-2 in treatment of SLE patients from north Chinese SLE patients (randomized controlled trials, RCTs) (42, 43, 149–154). SLE patients received three cycles of human IL-2 treatment (1 million IU every other day for 2 weeks, followed by a 2-week break), showing reduced SLEDAI score, less myositis, fever, alopecia, vasculitis, arthritis, oral ulcer, low rate of infection, decreased number of CXCR5^+^PD-1^+^CCR7^lo^ Tfh cells, CCR6^+^CXCR3^−^CCR4^+^CCR7^lo^ Th17 cells, and CD4^−^CD8^−^ αβ T cells at the 12th week, and an elevated proportion of CXCR5^+^PD-1^lo^ Treg cells and CXCR5^+^PD-1^hi^ Treg cells and ratios of CXCR5^+^PD-1^lo^ Treg/Tfh, CXCR5^+^PD-1^hi^ Treg/Tfh, CXCR5^+^PD-1^lo^ Treg/Th17, and CXCR5^+^PD-1^hi^ Treg/Th17 cells (43, 150, 152). After treatment at the 12th week, SLE patients who had SRI-4 were recognized as good response (GR); otherwise, the patients were considered as poor response (PR) (130). As compared with the PR group of patients, the GR group of patients was related to a low ratio of Treg cells and a ratio of Treg/Th17 cells at baseline (152). When dividing SLE patients into patients with a low proportion of Treg cells and patients with a high proportion of Treg cells, SLE patients with a low proportion of Tregs had higher probability of achieving GR to IL-2 treatment at the 12th week. After following up IL-2-treated SLE patients at the 24th week, the SRI-4 response rate was 65.52%, and 53.85% SLE patients had complete remission (153). Interestingly, comparing the advantage of prednisone treatment of SLE patients and prednisone+IL-2 treatment of SLE patients, it was found that 36.36% patients had a reduced proportion of Treg cells upon prednisone treatment, and prednisone treatment downregulated the Foxp3 expression in CD4^+^ T cells and the expression of CD39, ICOS, Bcl-2, and STAT5 in CD4^+^Foxp3^+^Treg cells (149). Prednisone+IL-2 treatment did not downregulate the number of Treg cells whereas it downregulated the SLEDAI score, upregulated the expression of C3 and C4, and improved rash and fever (149). For IL-2 treatment of LN patients, low-dose human IL-2 in treatment of active LN patients improved nephritis, achieved a higher remission rate as compared with placebo, and promoted the proliferation of Treg cells (154). Administration of human IL-2 in refractory LN patients reported that most patients had reduced proteinuria, along with decreased urine erythrocytes and anti-dsDNA production, elevated expression of C3 and C4, and high percentage of Treg cells (151). Furthermore, two studies discussed the role of human IL-2 for treating SLE patients from Northwest China (RCTs). The first study used a dose of 0.5 million IU/day for SLE patients with infection (5-day course), revealing that low-dose IL-2 treatment upregulated the number of total T cells, B cells, CD4^+^ T cells, CD8^+^ T cells, Th1, Th17, and Treg cells, which were similar to those in healthy controls (155). Another study discussed combined treatment of IL-2 (1 million IU/day, 3–5 days/4 weeks) and rapamycin (0.5 mg/day, once every other day) for refractory SLE patients (24 weeks’ treatment), reporting that the number of Treg cells at the 12th and 24th weeks was increased, and the SLEDAI score and the ratio of Th17/Treg cells were reduced (156). The findings were partly similar to a study from eastern China SLE patients, where SLE patients treated with 1 million IU/day IL-2 (every second day for 2 weeks plus a 2-week break, three cycles) revealed a lower frequency of CD4^+^CXCR5^+^ cells and increased ratio of Tfr/Tfh cells (RCTs) (157). Moreover, two studies on German SLE patients discussed 1.5 million IU/day of human IL-2 for treatment of SLE patients (RCTs). The first one included 5 consecutive days of treatment for refractory SLE patients, showing that the number of CD3^+^CD4^+^Foxp3^+^CD127^lo^ Treg cells and expression of CD25 in Treg cells were increased (143). The second one included 5 consecutive days/week of treatment for active SLE patients, followed by weekly injection for 12 weeks (158). The authors found a higher proportion of patients achieving SRI-6 response and SRI-8 response compared with patients treated with placebo, and more patients had clinical remission, such as resolved arthritis, rash, mucosal ulcers, alopecia, and increased percentage of CD3^+^CD4^+^Foxp3^+^CD127^lo^CD25^hi^ Treg cells and CD3^-^CD56^+^ NK cells (158).

**Table 2:** Studies discussed IL-2 treatment in lupus.

Year	Author	Country/ethnicity	Treatment	Main results	Reference
2015	Spee-Mayer et al.	German	1.5 million IU/day, 5 day course	Refractory SLE patients showed high number of CD3^+^CD4^+^Foxp3^+^CD127^lo^ Treg cells and expression of CD25 in Treg cells.	130^a^
2016	He et al.	North China	1 million IU every other day for 2 weeks, followed by a 2-week break, 3 cycles	SLE patients reported reduced SLEDAI score, less fever, alopecia, arthritis, decreased number of CD4^-^CD8^-^ αβ T cells.	143^a^
2019	Zhao et al.	Northwest China	1 million IU/day, 3–5 days/4 weeks, 6 cycles	Combined treatment of IL-2 and rapamycin for refractory SLE patients upregulated number of Treg cells, decreased SLEDAI score.	145^a^
2019	Shao et al.	North China	1 million IU every other day for 2 weeks, followed by a 2-week break, 3 cycles	Active LN patients improved nephritis, achieved higher remission rate and promoted proliferation of Treg cells.	142^a^
2020	He et al.	North China	1 million IU every other day for 2 weeks, followed by a 2-week break, 3 cycles	Most SLE patients had complete remission, no serious infection and expanded Treg cells, NK cells.	141^a^
2021	Zhou et al.	North China	1 million IU every other day for 2 weeks, followed by a 2-week break, 3 cycles	SLE patients had reduced rate of infection (bacterial infection, virus infection).	137^a^
2021	Zhang et al.	North China	1 million IU every other day for 2 weeks, followed by a 2-week break, 3 cycles	Most of refractory LN patients showed reduced proteinuria, urine erythrocytes, anti-dsDNA production, and elevated expression of C3, C4.	138^a^
2021	Miao et al.	North China	1 million IU every other day for 2 weeks, followed by a 2-week break, 3 cycles	SLE patients revealed low SLEDAI score, less myositis, fever, alopecia, vasculitis, arthritis, oral ulcer, and decreased number of CXCR5^+^PD-1^+^CCR7^lo^ Tfh cells, CCR6^+^CXCR3^-^CCR4^+^CCR7^lo^ Th17 cells.	139^a^
2021	Liang et al.	East China	1 million IU/day, every second day for 2 weeks plus a 2-week break, 3 cycles	SLE patients had low frequency of CD4^+^CXCR5^+^ cells, increased ratio of Tfr/Tfh cells.	146^a^
2022	Miao et al.	North China	1 million IU every other day for 2 weeks, followed by a 2-week break, 3 cycles	SLE patients had lower proportion of Treg cells at baseline will show better response to IL-2 treatment.	140^a^
2022	Zhang et al.	Northwest China	0.5 million IU/day, 5 day course	Treatment upregulated number of total T cells, B cells, CD4^+^ T cells, CD8^+^ T cells, Th1, Th17, Treg cells in SLE patients.	144^a^
2022	Humrich et al.	German	1.5 million IU/day, 5 consecutive days/week, 12 cycles	More SLE patients achieved SRI-6, SRI-8 response, and had increased percentage of CD3^+^CD4^+^Foxp3^+^CD127^lo^CD25^hi^ Treg cells, CD3^-^CD56^+^ NK cells.	147^a^
2023	Zhou et al.	North China	1 million IU every other day for 2 weeks, followed by a 2-week break, 3 cycles	Combined treatment of prednisone+IL-2 for SLE patients downregulated SLEDAI score, increased expression of C3, C4, and improved rash, fever.	136^a^
2015	Ohl et al.	German	25 ng/g bodyweight every 5 days, 5 times	FVB/Fas^-/-^ lupus-like mice inhibited lymphadenopathy, splenomegaly, upregulated number of Treg cells, and IFNγ expression.	154^b^
2019	Rose et al.	German	25 ng/g bodyweight every day, 5 day course, or 25 ng/g bodyweight every 4 days, 6 times	Both treatment for (NZB×NZW)F1 mice upregulated CD4^+^Foxp3^+^ Treg cells, and suppressed proportion of CD4^+^Foxp3^-^CD44^hi^ cells.	152^b^
2019	Taylor et al.	North American	2 μg every 5 days, 5 times	(NZB×NZW)F1 mice had more Treg cells, and low mean arterial pressure.	153^b^
2021	Liang et al.	China	30,000 IU/day, 1 week, or 2 week, or 4 weeks	(NZB×NZW)F1 mice showed high percentage of B220^-^CD4^+^Foxp3^+^ Treg cells, reduced CD4^+^Foxp3^-^CD44^+^ T cells after 1 week treatment. There were expanded B220^-^CD4^+^Foxp3^+^ Treg cells and suppressed germinal center B-cell formation after 2 week’s treatment. Low expression of anti-dsDNA, C3, and IgG1 alleviated renal histopathology after 4 weeks’ treatment.	146^b^
2023	Cao et al.	China	30,000 IU/day, 1 week	MRL/lpr mice had lymph nodes shrunk, ameliorated glomerulonephritis, reduced expression of anti-dsDNA, IFNγ, and urine protein after 1 week treatment and were returned to the pre-treatment level 4 weeks later.	134^b^

Both SLE patients and lupus mice models were treated with low-dose IL-2.

a Human.

b Mouse models.

Treg cell, regulatory T cell; SLEDAI, systemic lupus erythematosus disease activity index; LN, lupus nephritis; NK cell, natural killer cell; Tfh cell, follicular T helper cell; Tfr, follicular regulatory T cell; SRI-6 response, SLEDAI-2K responder index-6.

Similarly, some studies with animal models clarified the role of IL-2 in SLE pathogenesis and blocking lupus development by IL-2 treatment. Pristane-induced lupus mice treated with L72-FSY ameliorated disease severity, evidenced by decreased expression of ANA, anti-RNP, anti-Sm antibodies, frequencies of CD8^+^ T, Tfh cells, and germinal center B cells, improvement of glomerulonephritis, renal inflammation, tubular degeneration, and less infiltration of mononuclear cells in the lung (142). When BDF1 lupus-like mice were treated with IL-2-loaded poly lactic-co-glycolic acid nanoparticles (IL-2-loaded NPs) targeted to CD3 T cells, the number of immature CD24^+^IgM^+^CD21^int/lo^ transitional 2 cells, mature follicular B cells, and Tfh cells was reduced (159). Similarly, lupus mice treated with IL-2-loaded NPs targeted to Tfh cells showed decreased number of Tfh cells, low serum levels of IgG1 and IgG2a, downregulated proteinuria, and extended survival (159). These were confirmed in other NPs, by which BDF1 lupus-like mice treated with anti-CD2/CD4 antibody-coated NPs encapsulating IL-2 will lead to a higher number of CD4^+^ and CD8^+^ Treg cells, decreased expression of anti-dsDNA, proteinuria, and preserved glomeruli (113). Injection of complex of IL-2 and anti-IL-2 monoclonal antibody into (NZB×NZW)F1 mice may lead to expansion of CD4^+^CD25^+^Foxp3^+^ Tregs in kidneys and spleen, along with reduced renal infiltration of T cells, B cells, reduced proteinuria, improved glomerular and tubular injury, vasculitis scores, and less renal deposition of IgG and C3 (160). MRL/lpr mice injected with adeno-associated virus with IL-2 (AAVIL-2) led to decreased mononuclear cell infiltration (for instance, IL-17-producing CD3^+^CD4^−^CD8^−^ T cells) in kidneys, skin, and lung and increased the percentage of CD4^+^CD25^+^Foxp3^+^ Treg cells in kidneys (161). For low-dose IL-2 treatment in lupus mice, MRL/lpr mice injected with IL-2 (30,000 IU/day for 1 week) reported that lymph nodes were shrunk and the expressions of anti-dsDNA, IFNγ, IL-10, urine protein, and creatinine were inhibited after 1 week treatment and were returned to the pretreatment level 4 weeks later (147). Histologic analysis showed that 1-week treatment significantly ameliorated glomerulonephritis, interstitial nephritis, and infiltration of lymphocytes surrounding blood vessels and downregulated glomerular C3 deposition and the proportion of Treg cells in spleen, whereas the pathogenic changes were similar to the pretreatment level after a 4-week treatment (147). This was partly demonstrated in (NZB×NZW) F1 mice, where the mice injected with IL-2 (30,000 IU/day for 1 week) expanded B220^−^CD4^+^Foxp3^+^ Treg cells and inhibited CD4^+^Foxp3^−^CD44^+^ T cells, CD4^+^Bcl-6^+^CXCR5^+^ Tfh cells, and CD4^+^Foxp3^+^Bcl-6^+^CXCR5^+^ Tfr cells (157). After 2 weeks’ treatment, the mice also expanded B220^-^CD4^+^Foxp3^+^ Treg cells and suppressed germinal center B-cell formation, and there were lower expressions of anti-dsDNA, C3, IgG1, IgG2a, IgG2b, and IgG3, alleviated renal histopathology such as reduced mesangial matrix expansion, and reduced urinary albumin after 4 weeks’ treatment (157). (NZB×NZW) F1 mice injected with IL-2 (25 ng/g bodyweight every day, 5 consecutive days) showed more CD4^+^Foxp3^+^ Treg cells, and IL-2 treatment (25 ng/g bodyweight every 4 days, six times) inhibited the proportion of CD4^+^Foxp3^−^CD44^hi^ cells, CD69^+^ cells, and Ki67^+^ cells (162). (NZB×NZW) F1 mice treated with IL-2 (2 μg every 5 days, five times) expanded Treg cells in the spleen and kidney and lowered the mean arterial pressure (163). FVB/Fas^−/−^ lupus-like mice had lymphadenopathy, splenomegaly, high proportion of CD4^−^CD8^−^ T cells, IFNγ-producing T cells, and low proportion of Treg cells, and addition of IL-2 (25 ng/g bodyweight every 5 days, five times) restored the number of Treg cells and upregulated IFNγ expression (164). On the contrary, (NZB×NZW)F1 mice administrated anti-IL-2 antibody downregulated the percentage of CD4^+^CD25^+^Foxp3^+^ Treg cells in the lymph nodes and spleen, led to acceleration of nephritis, and increased mortality (165), and MRL/lpr mice administrated anti-IL-2 antibody promoted IL-17^+^ γδ T-cell proliferation (148).

## 5 Conclusion

Th1 cells and related transcription factor T-bet and inflammatory cytokines including IFNγ, TNFα, IL-2, IL-18, TGF-β, and IL-12 are important in inflammatory responses such as autoimmunity development (166, 167). To date, cumulative evidence from both *in vitro* and *in vivo* studies has discussed roles of the Th1 family in lupus pathogenesis either from lupus patients or from animal models. All of the findings indeed imply essential roles of the Th1 family in lupus. Interestingly, current updated advancements have comprehensively clarified more about the new hypothesis of IL-2 in SLE treatment and T-bet involved in lupus development by regulating B cells. This review is helpful for better understanding the Th1 family in lupus and may give more clues for targeting the Th1 family including IL-2 in the future in SLE treatment. However, this study has some limitations. First, many studies have discussed IL-18 and TNFα gene polymorphisms and SLE risk. However, all the available evidence only discussed the association of IL-18 and TNFα gene polymorphisms and SLE susceptibility. How mutated IL-18 and TNFα gene polymorphisms regulate the mRNA expression of IL-18 and TNFα and then be involved in lupus pathogenesis needs discussion. Similarly, SLE is a chronic inflammatory disorder. Some studies have revealed the role of genetic mutation interacting with environment factors in SLE pathogenesis. Current studies about Th1 family genes did not discuss genetic mutation interacting with environment factors or gene–gene interactions in SLE pathogenesis. Second, targeting IL-2 in both SLE patients and animal models seems potential and meaningful; however, current studies do not have large sample sizes and multicenters. Hopefully, in the future, more studies with different countries and larger sample sizes will be conducted globally to confirm the role of low-dose IL-2 in treatment of SLE. Moreover, different doses of IL-2 in SLE treatment and different observational course have made inconsistent results based on current studies, for instance, in China. Therefore, at least, national population-based study in China needs to discuss IL-2 treatment in SLE patients. Moreover, exploring the diversity of patient populations would shed light on the applicability of low-dose IL-2 treatment across various demographics, disease stages, and ethnic groups. Third, molecular mechanisms of cytokines such as IL-18, TGF-β, and IL-12 involved in SLE pathogenesis or development have not been comprehensively discussed, where limited functional studies have discussed their roles in SLE. Thus, how they contribute to SLE or inhibit SLE needs more studies to clarify their role in SLE. Maybe, targeting these inflammatory cytokines in SLE may look like to targeting IL-2 in SLE in the future. Although much remains to be discussed, it is now accepted that the Th1 family is critical in SLE pathogenesis.

## Author contributions

Y-YT: Project administration, Writing – review & editing. D-CW: Writing – review & editing. Y-YC: Writing – review & editing. W-DX: Writing – review & editing. A-FH: Supervision, Writing – review & editing.
